# Supplementary material for: Tailoring photoluminescence of WS2-microcavity coupling devices in broad visible range
Source: Nanophotonics. 2023 Jan 24;12(4):753–60. doi: 10.1515/nanoph-2022-0705 (PMC11636489; doi:10.1515/nanoph-2022-0705)
Supplement: Supplementary file 1 — Supplementary Material Details [file j_nanoph-2022-0705_suppl_001.pdf]

Supplementary Material

Tailoring Photoluminescence of WS_2-Microcavity Coupling Devices in Broad Visible Range

Le-Yi Zhao[1], Hai Wang[2,], Tian-Yu Liu[2], Fang-Fei Li[1], Qiang Zhou[1,]* and Hai-Yu Wang[2,]**

1. Synergetic Extreme Condition High-Pressure Science Center, State Key Laboratory of Superhard Materials, College of Physics, Jilin University, Changchun 130012, China.

2. State Key Laboratory of Integrated Optoelectronics, College of Electronic Science and Engineering, Jilin University, 2699 Qianjin Street, Changchun 130012, China.

Corresponding Author Emails: wanghai03@jlu.edu.cn, zhouqiang@jlu.edu.cn, haiyu_wang@jlu.edu.cn

1. Comparison of reflection spectra of bare cavities and WS_2-microcavity devices.

	L/2 (nm)
**device a**	40
**device b**	42.5
**device c**	45
**device d**	47.5
**device e**	50
**device f**	52.5
**device g**	55
**device h**	60

Table S1. The parameter details of the cavity length (L) in devices a-h.

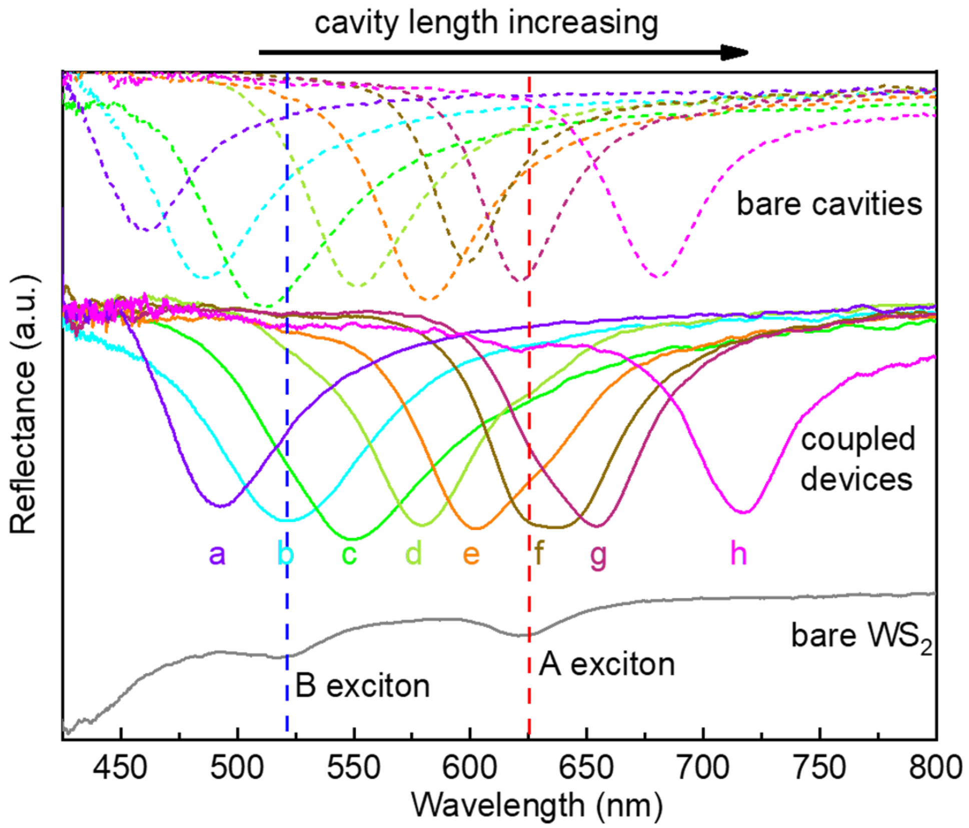

Figure S1. Comparison of reflection spectra of bare cavities and WS_2-microcavity devices.

2. Comparison of Raman spectra of bare WS_2 and WS_2-microcavity devices.

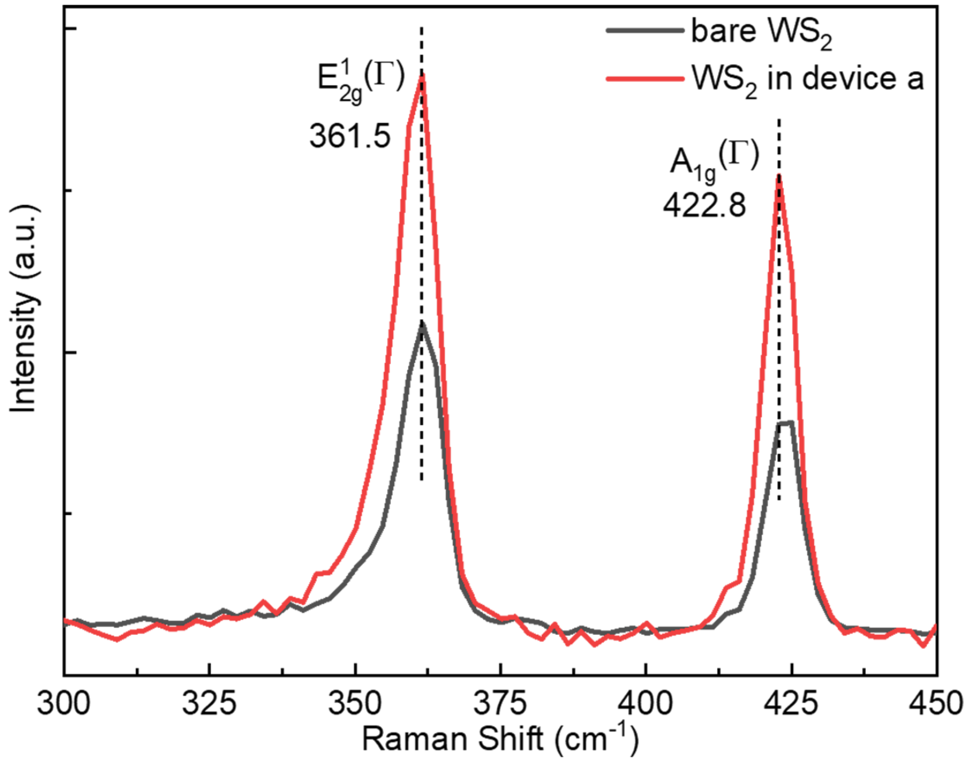

Figure S2. Comparison of Raman spectra of bare monolayer WS_2 on SiO_2/Ag film and monolayer WS_2 embedded in the microcavity.

The quality of the monolayer WS_2 embedded in cavity have been demonstrated by Raman spectra as shown in Figure S1. The two characteristic Raman peaks, E_{2g}^{1} and A_{1g}, represent the in-plane and out-of-plane vibrational modes, respectively. The frequency separation between E_{2g}^{1} (361.5 cm^{-1}) and A_{1g} (422.5 cm^{-1}) phonon modes of WS_2 is 61.0 cm^{-1}, meeting the standard of monolayer WS_2. Additionally, the peaks of the Raman spectra in bare monolayer WS_2 on SiO_2/Ag film are almost the same as that in the microcavity, which indicates that the evaporation of the upper SiO_2 film does not cause the deformation of monolayer WS_2.

3. Modulation on the reflection spectra of devices e-g.

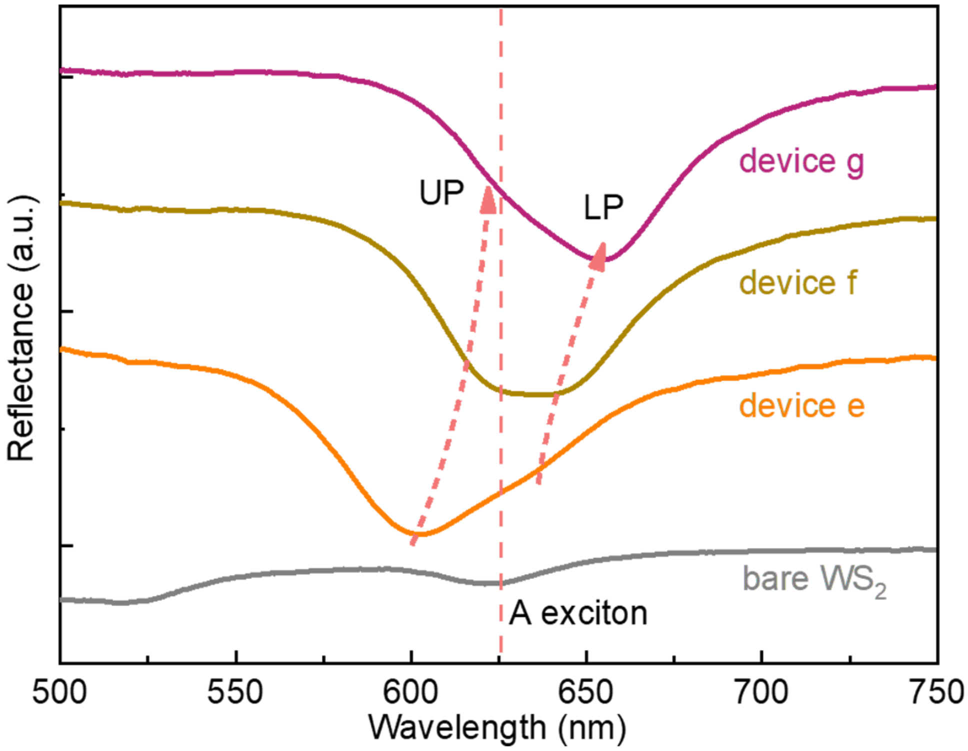

Figure S3. Reflection spectra of devices e-g and bare monolayer WS_2 on SiO_2/Ag film.

4. Modulation on the TA spectra of devices a-c and devices e-h.

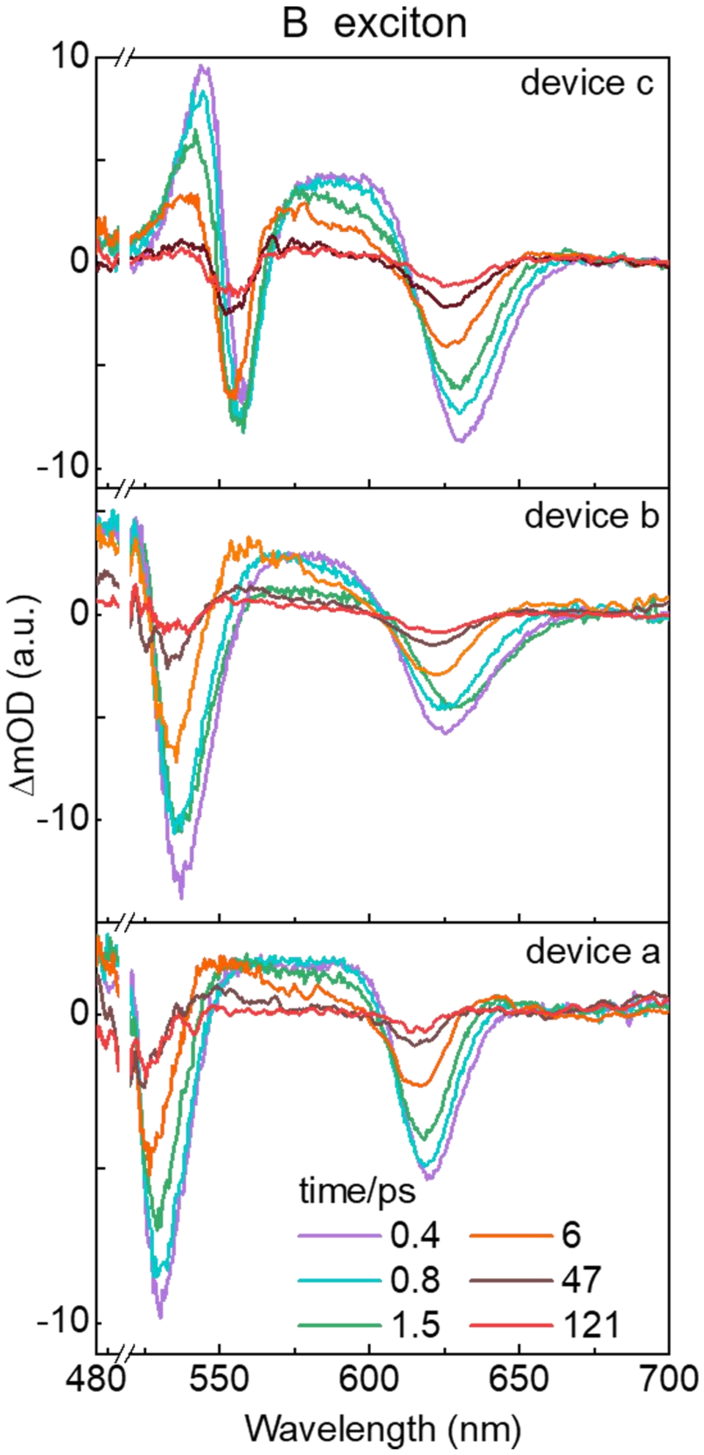

Figure S4. TA spectra of devices a-h at different delay times (0.4 ps, 0.8 ps, 1.5 ps, 6 ps, 47 ps, 121 ps). The excitation wavelength is at 500 nm.

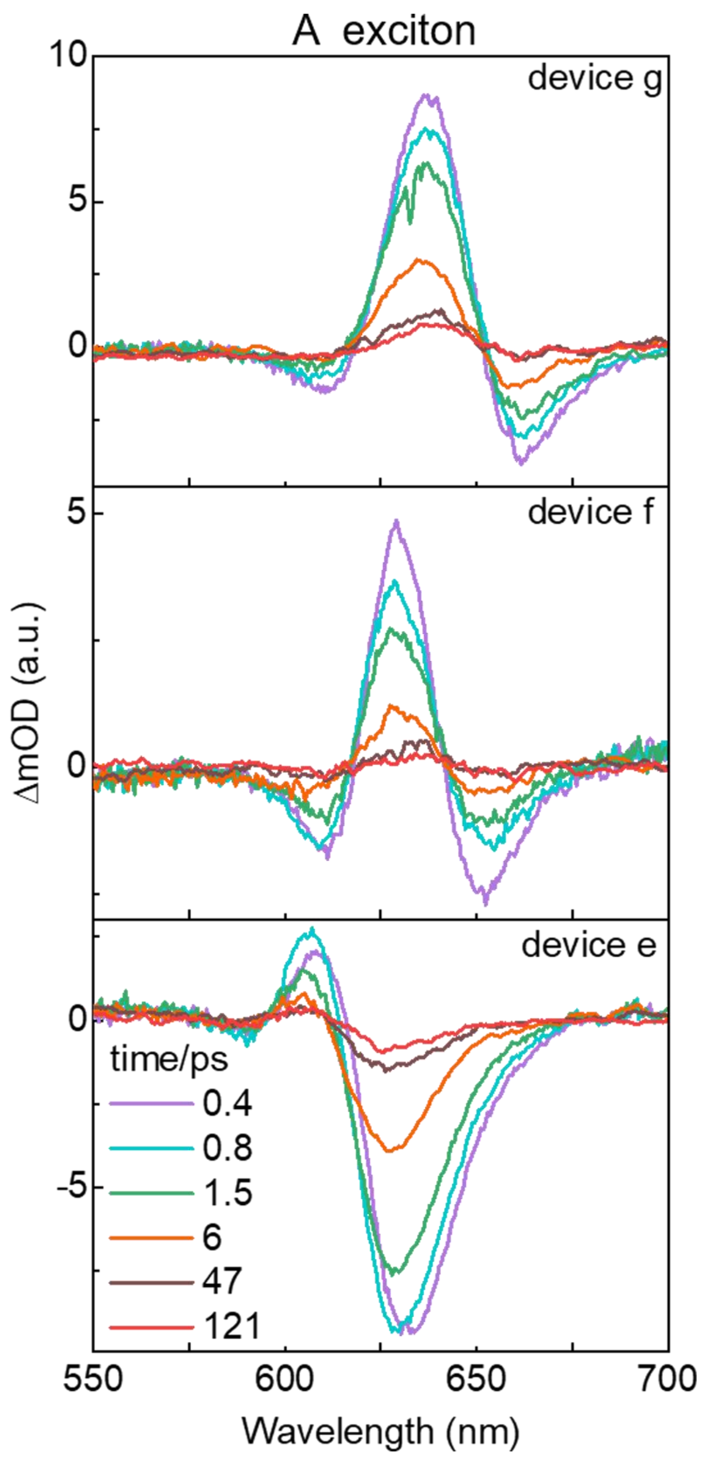

Figure S5. TA spectra of devices e-f at different delay times (0.4 ps, 0.8 ps, 1.5 ps, 6 ps, 47 ps, and 121 ps). The excitation wavelength is at 500 nm.

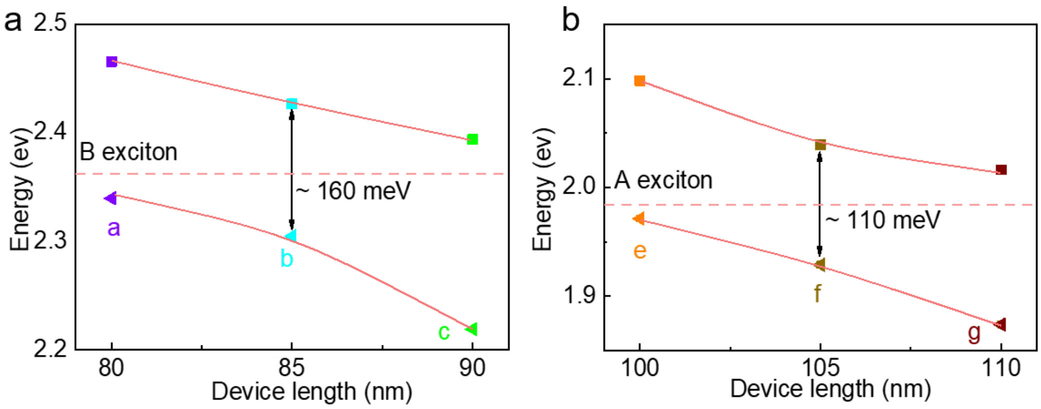

Figure S6. The dispersion curves of the UP and LP branches in devices a-c (a) and devices e-g (b).

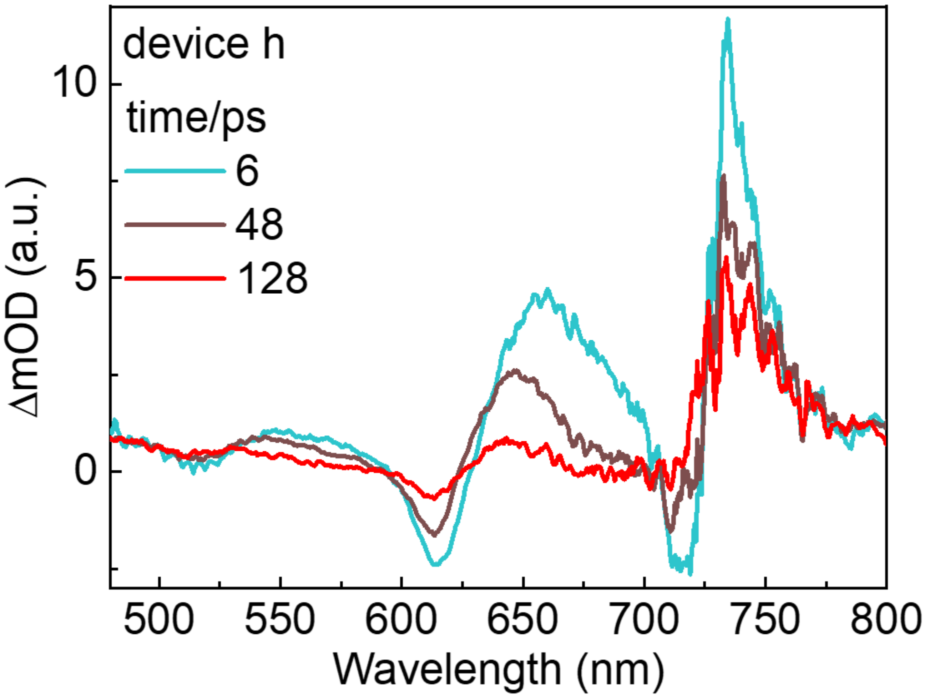

Figure S7. TA spectra of device h at different delay times (6 ps, 48 ps, and 128 ps).
