# Supplementary material for: Tailoring photoluminescence of WS2-microcavity coupling devices in broad visible range
Source: Nanophotonics. 2023 Jan 24;12(4):753–60. doi: 10.1515/nanoph-2022-0705 (PMC11636489; doi:10.1515/nanoph-2022-0705)
Supplement: Supplementary file 2 — Supplementary Material Details [file j_nanoph-2022-0705_suppl_002.docx]

**Article type: Full Paper**

**Tailoring Photoluminescence of** **WS_2_-Microcavity Coupling Devices in Broad Visible Range**

Le-Yi Zhao^1^, Hai Wang^2,^*, Tian-Yu Liu^2^, Fang-Fei Li^1^, Qiang Zhou^1,^* and Hai-Yu Wang^2,^*

1. Synergetic Extreme Condition High-Pressure Science Center, State Key Laboratory of Superhard Materials, College of Physics, Jilin University, Changchun 130012, China.

2. State Key Laboratory of Integrated Optoelectronics, College of Electronic Science and Engineering, Jilin University, 2699 Qianjin Street, Changchun 130012, China.

Corresponding Author Emails: [wanghai03@jlu.edu.cn](mailto:wanghai03@jlu.edu.cn), [zhouqiang@jlu.edu.cn](mailto:zhouqiang@jlu.edu.cn), [haiyu_wang@jlu.edu.cn](mailto:haiyu_wang@jlu.edu.cn)

**Keywords:** photoluminescence enhancement, Rydberg state, optical microcavity, strong coupling, transient absorption, [transition metal dichalcogenides](https://www.webofscience.com/wos/alldb/general-summary?queryJson=%5B%7B%22rowBoolean%22:null,%22rowField%22:%22AK%22,%22rowText%22:%22%5C%22transition%20metal%20dichalcogenides%5C%22%22%7D%5D&eventMode=oneClickSearch)

# Abstract

Most of the previous TMDC-photon coupling devices were mainly based on A exciton due to its high oscillator strength and large exciton binding energy. Less effort has been focused on the modulation of the emission of B exciton and Rydberg states in TMDCs, especially in monolayer WS_2_. Here, we demonstrate that the photoluminescence (PL) emission of WS_2_-microcavity coupling devices can be tailored in a broad visible wavelength range (490 nm – 720 nm). In contrast to the intrinsic PL emission of monolayer WS_2_, 25-fold enhanced B exciton emission and significant PL emission from the 2s Rydberg state can be observed. From the transient absorption (TA) measurements, the strongly coupled hybrid states based on B exciton can be remarkably fingerprinted. Furthermore, the strongly enhanced PL emission from the coupled B exciton has been demonstrated due to the strongly increased lower polariton (LP) state population and the internal conversion pathway being blocked in the strong coupling regime. Besides, the remarkable PL emission from the 2s Rydberg state is also revealed and confirmed by the additional ground state bleaching signal in TA spectra. These physical mechanisms about tailoring the PL emission in low dimensional TMDCs can provide significant references for constructing highly efficient optoelectronic devices.


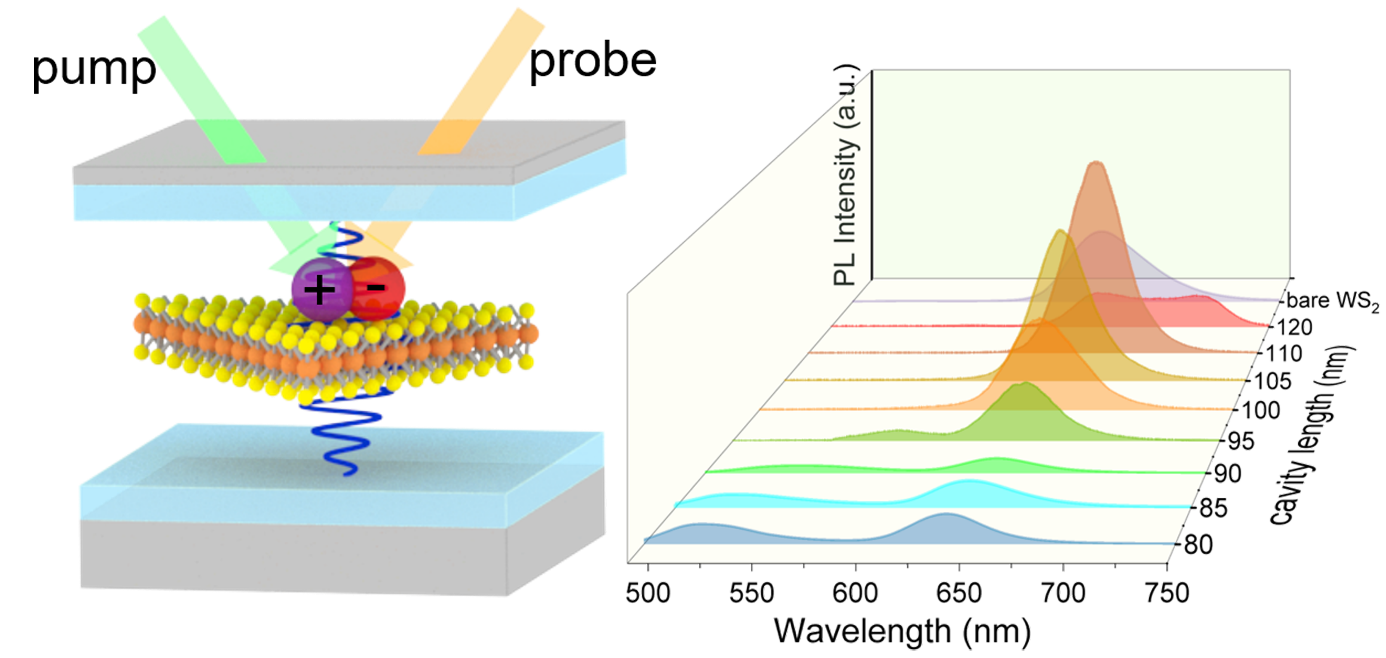


# 1. Introduction

As direct band gap semiconductor in the visible range, monolayer transition metal dichalcogenides (TMDCs) possess efficient light emission even at room temperature [1-4]. Due to relatively low defect density, the photoluminescence quantum yield (PLQY) of A exciton in monolayer WS_2_ has been measured as high as 6%, which is the highest among all kinds of monolayer TMDCs [5]. Furthermore, the strong spin-orbit coupling in monolayer WS_2_ leads to energy splitting in the valence band maximum, resulting in the observation of another relatively weak B exciton resonance [6]. The valence band splitting between the A and B excitons of monolayer WS_2_ (~ 400 meV) is larger than the other monolayer TMDCs (e.g. ~150 meV in monolayer MoS_2_) due to the larger mass of tungsten atoms [6]. Therefore, the emission of A and B excitons in monolayer WS_2_ can be modulated in a much wider spectral range. In addition, at the higher energy side of A exciton, there are a series of other internal exciton states that mimic the Rydberg states in the hydrogen atom arising from the strong Coulomb interaction [7]. Although the oscillator strength of these excited Rydberg states is even much weaker than that of B exciton, PL emission from these states have been well confirmed in the previous experiments [7-9]. Thus, achieving modulation on these Rydberg states with remarkable properties would significantly enrich the excitonic physics in 2D semiconductors [7, 9-14]. Meanwhile, combined with the modulate on the emission from A and B excitons, monolayer WS_2_ provides an attractive platform for realizing optoelectronic devices operating at multiple wavelengths.

Integrating TMDCs into resonant cavities such as conventional Fabry-Perot microcavity or plasmonic resonators is the most common strategy to promote exciton emission. By coupling optical resonators with different excitonic states in monolayer WS_2_, various coupling systems from weak to strong coupling regimes can be constructed [15-17]. In weak coupling regime, the intrinsic energy level of the material remains unchanged, where the spontaneous emission rate of the excitons can be greatly modified by the Purcell effect, resulting in the enhancement of the PL emission [18-20]. On the other hand, in strong coupling regime, the rate of energy exchange between the light and the excitons is faster than any other decoherent process, which can result in the generation of photon-exciton hybrid states, called exciton-polaritons [21-28]. As half-light half-matter particles, exciton-polaritons exhibit great demonstrations such as low threshold polariton lasing and strong valley-dependent polarized emission [21, 29-31]. While, most of the previous works were focused on strong coupling with A exciton of monolayer TMDCs, due to its high oscillator strength and large exciton binding energy [9, 24, 31-33]. Achieving the coupling devices based on B exciton in TMDCs needs to increase the number of the TMDC layers [34] or enhance the light field in plasmonic nanocavity [35, 36]. The oscillator strength of Rydberg states is even much weaker than that of B exciton. Therefore, less effort has been focused on the modulation of the emission of B exciton and Rydberg states in monolayer TMDCs, especially in monolayer WS_2_.

In this work, we demonstrate that the PL emission of the A/B exciton and Rydberg state of monolayer WS_2_ can be efficiently modulated in a wide spectral range (490 nm – 720 nm) by embedding it into Ag microcavities with tunable cavity resonances. Furthermore, by the transient absorption (TA) measurements, strongly coupled hybrid states formed by the A/B exciton and the cavity photons are clearly shown. In particular, with respect to the intrinsic emission from bare WS_2_, significantly enhanced PL emission from the lower polariton (LP) states coupled with B exciton can be [attributed to](javascript:;) the strongly increased LP state population and also associated with the internal conversion pathway being blocked in the strong coupling regime. Moreover, when the cavity mode is tuned to the higher energy side of the A exciton, an additional bleaching signal in the TA spectra is observed, which is regarded as the cavity resonance enhanced 2s Rydberg state, leading to the remarkable PL emission peak at the 2s Rydberg state.

# 2. Result and discussion

## 2.1. Static measurements

The WS_2_-microcavity coupling devices were formed by embedding triangular monolayer WS_2_ flakes into the center of the Ag microcavity as illustrated in **Figure 1**a. The thickness of the Ag film at the bottom and the top of the WS_2_-microcavity devices is 100 nm and 20 nm, respectively. The thickness of the top half SiO_2_ film (L/2) was the same as that of the bottom half SiO_2_ film, varying from 40 nm to 60 nm (the parameter details of devices a-h was shown in **Table S1**). The pure cavity mode can be tuned from 460 nm to 680 nm (dashed lines plotted at the top of **Figure S1**). The monolayer WS_2_ flakes in the center of the microcavity were transferred onto the bottom half SiO_2_ film layer via a wet transfer method (see Methods for details). The optical microscope image of the WS_2_-microcavity coupled device is shown in Figure 1b. Before evaporating the upper Ag film, the bare monolayer WS_2_ in the open cavity can be regarded as the control sample, whose reflection spectrum is plotted in Figure S1 (gray solid line). The two peaks at 620 nm and 520 nm can be assigned to the A and B exciton, respectively. Then, after evaporating the upper Ag layer, compared with the pure microcavity, a noticeable red-shifted of the reflection spectra can be found by a first look (solid lines at the bottom of Figure S1). However, by further carefully analyzing these reflection spectra, combined with the PL emission spectra, the cavity-induced modulation on the A, B, and Rydberg excitons can be revealed more clearly.

First, as shown in Figure 1c, the modulation of B exciton was examined in the WS_2_-microcavity coupled devices a-c, in which the cavity mode swept over the B exciton resonance. It is found that the reflection spectra of the coupled devices are weakly disturbed, which can be attributed to the weak oscillator strength of the B exciton. Nevertheless, the modulation of the PL emission can be clearly observed as shown at the bottom of Figure 1c. As a reference, the intrinsic PL from monolayer WS_2_ in the open cavity was first measured and exhibited a dominated A exciton emission at 642 nm, while the emission from B exciton at 525 nm was extremely weak (gray line in Figure 1c). In sharp contrast, a significantly enhanced emission around B exciton was presented in the coupled devices a-c, which possess an enhancement factor of 25, 17, and 14 respectively. The largest PL enhancement factor is observed in device a, which can be caused by the good overlap between the cavity mode and the B exciton resonance. Besides, the peak positions are also strongly modified in the coupled devices. The PL emission peak can be tuned as far as 545 nm in device c, which has the longest cavity mode of the three devices. Another it should be noted that the intensity of the PL emission of A exciton in devices a-c was weakened due to the confinement effect by the microcavity [16], while the peak position is almost unchanged. Thus, we can easily derive the PL emission originating from the A and B excitons featuring comparable intensity in the coupled devices a-c, which provides a good foundation for the development of white light-emitting diodes based on TMDCs [37].

Next, with the increase of the cavity length, the cavity mode redshift and sweep over the A exciton resonance of monolayer WS_2_. Due to the much stronger oscillator strength of the A exciton, the reflection spectra of the coupled devices d-h show a more pronounced modulation. Notably, in **Figure S3**, the reflection spectra of the devices e-g slightly split into the upper polariton (UP) and the LP hybrid branch, whose peaks with the anti-crossing behavior showed the evidence of strong coupling regime. In addition, with the cavity length increasing, the PL emission peak of the coupled devices also shows a red-shift from 643 nm to 656 nm, which is corresponding to the conventional strong coupled system where the emission is from the LP state [38, 39]. Moreover, by further increasing the cavity length, the shape of the PL emission from the coupled device h is more strongly modified. As the pink line shown in Figure 1d, a shoulder peak appeared at 720 nm, which shifts over 100 nm compared with the intrinsic A exciton emission.

Finally, a more interesting phenomenon can be found in device d, in which the cavity mode is tuned to the position between the A and B exciton resonance (green line in Figure 1d). In this case, a noticeable PL emission peak appeared at 578 nm on the higher energy side of the A exciton. Indeed, this emission peak is very close to the 2s Rydberg state of A exciton in the previous reports [13]. Thus, we suggest that emission from the excited Rydberg state of A exciton can be obviously enhanced by the Purcell effect when the cavity mode matches the Rydberg state resonance.


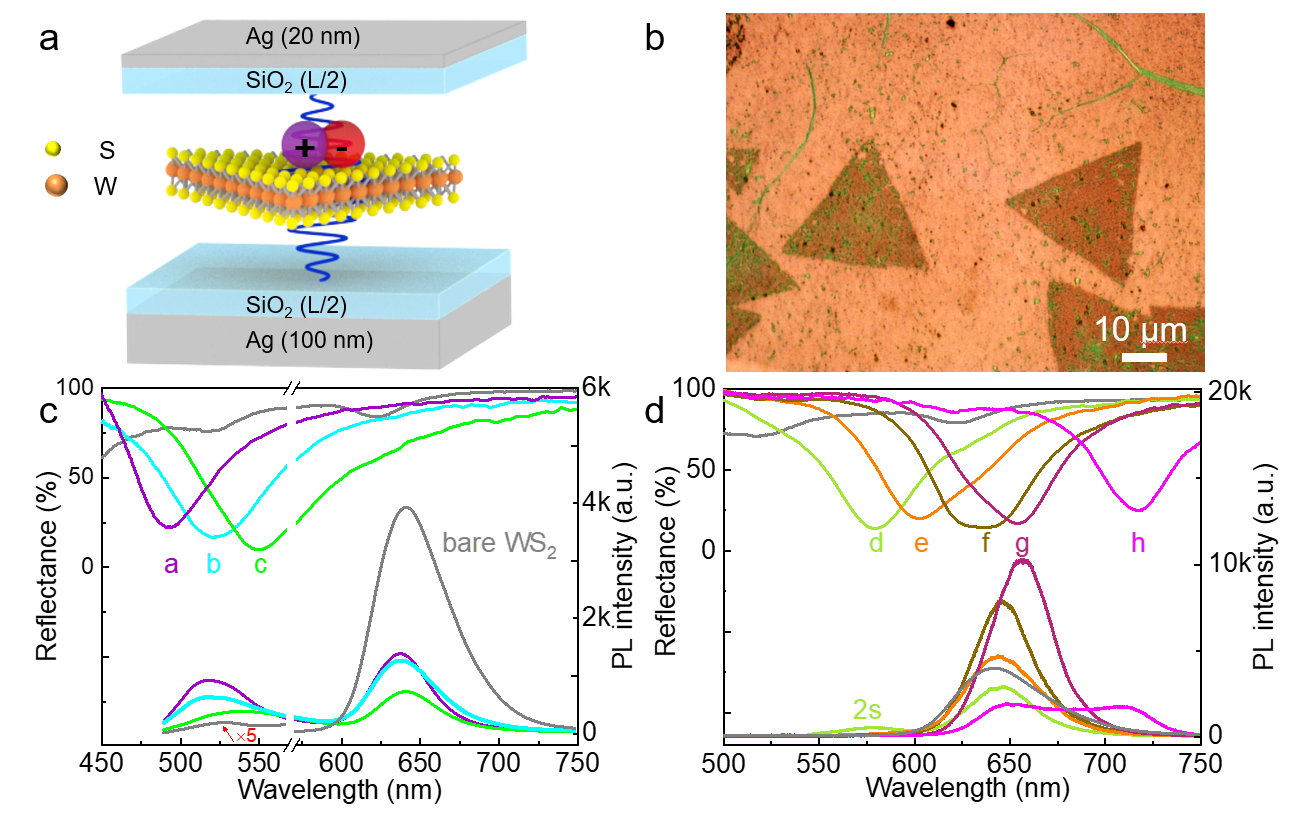


**Figure 1**. Modulation of the WS_2_-microcavity devices. (a) Schematic diagram of the WS_2_-microcavity device. (b) The optical image of WS_2_-microcavity device. (c) –(d) the reflection and PL spectra of WS_2_-microcavity devices. The excitation wavelength is at 473 nm.

Overall, through the cavity-induced modulation on the A, B, and Rydberg exciton states, the emission from the coupled devices can be flexibly tuned in the range of 490 to 750 nm, which covers most of the visible spectra range. Whereas, the mechanism of the cavity-induced modification on the PL emission, especially in the coupled devices with B exciton and Rydberg states is still unclear. To deeper explore the dynamics of the coupling effect in the WS_2_-microcavity devices, TA spectroscopy was further carried out.

## 2.2. Transient absorption experiments

The first series of TA experiments (see Methods for details) were performed by a 500 nm pump laser pulse on the WS_2_-microcavity coupled devices a-c to examine the modulation of B exciton. As a reference, the TA spectra of monolayer WS_2_ in the open cavity without the top Ag layer was first illustrated in **Figure 2**a. The plots show a dominated negative ground state bleaching (GSB) signal at 625 nm associated with the A exciton of monolayer WS_2_; while the GSB signal at 525nm associated with the B exciton is much weaker, which is consistent with the previous reports [40]. The positive signal at 650 nm was ascribed to the photon induced absorption. Then, the TA spectra of the WS_2_-microcavity coupled devices a-c were measured (see **Figure S4**). As a representative shown in Figure 2b, the TA spectra of device b showed very different characteristics with respect to the control sample. Especially in the B exciton region, A huge negative signal appears at 535 nm, which is possibly corresponding to the LP state. While the UP state is rarely present being it in the disturbed spectral region by the pump laser. Even though, from the less disturbed initial TA spectra at 0.4 ps (Figure 2c), a smaller GSB signal corresponding to the UP state can be found. With the cavity length increasing, both the UP and LP states show a trend of red shifting, which conformed to the signature of strong coupling: anti-crossing behavior. Thus, we identify that strong coupling regime can be achieved with B excitons in monolayer WS_2_. The dispersion of the UP and LP branches in devices a-c is plotted in Figure S6a, in which a Rabi splitting of ~160 meV can be measured. Moreover, the amplitude of the GSB signal of the LP states in coupled devices a-c shows a ~10-fold enhancement compared with that of bare B exciton states, which indicates that much more population present in the LP state. Hence, a large PL emission enhancement factor is expected in devices a-c.

On the other hand, such enhancement can be further explained by analyzing the dynamic process of the strong coupled system with B exciton. As shown in Figure 2d, the relaxation rates of the LP states in devices a-c are all significantly slower than that of bare B exciton. Indeed, when the strong coupled hybrid states are formed with high-energy excited state, the internal conversion pathway, that is the population flows from the high-energy to the low-energy excited state, can be blocked [40]. So different from bare monolayer WS_2_, in which the population in B exciton state would first rapidly relax to the lower energy level, namely A exciton state, and then to the ground state, the population in the LP state (coupled with B excitons) preferred to directly relax to the ground state without going through the A exciton state. At the same time, due to the population in the LP state being hugely increased as we have discussed above, hence the large PL emission enhancement factor relative to the LP states (coupled with B excitons) is expected in the coupled devices a-c, as we have observed in static PL emission measurements.


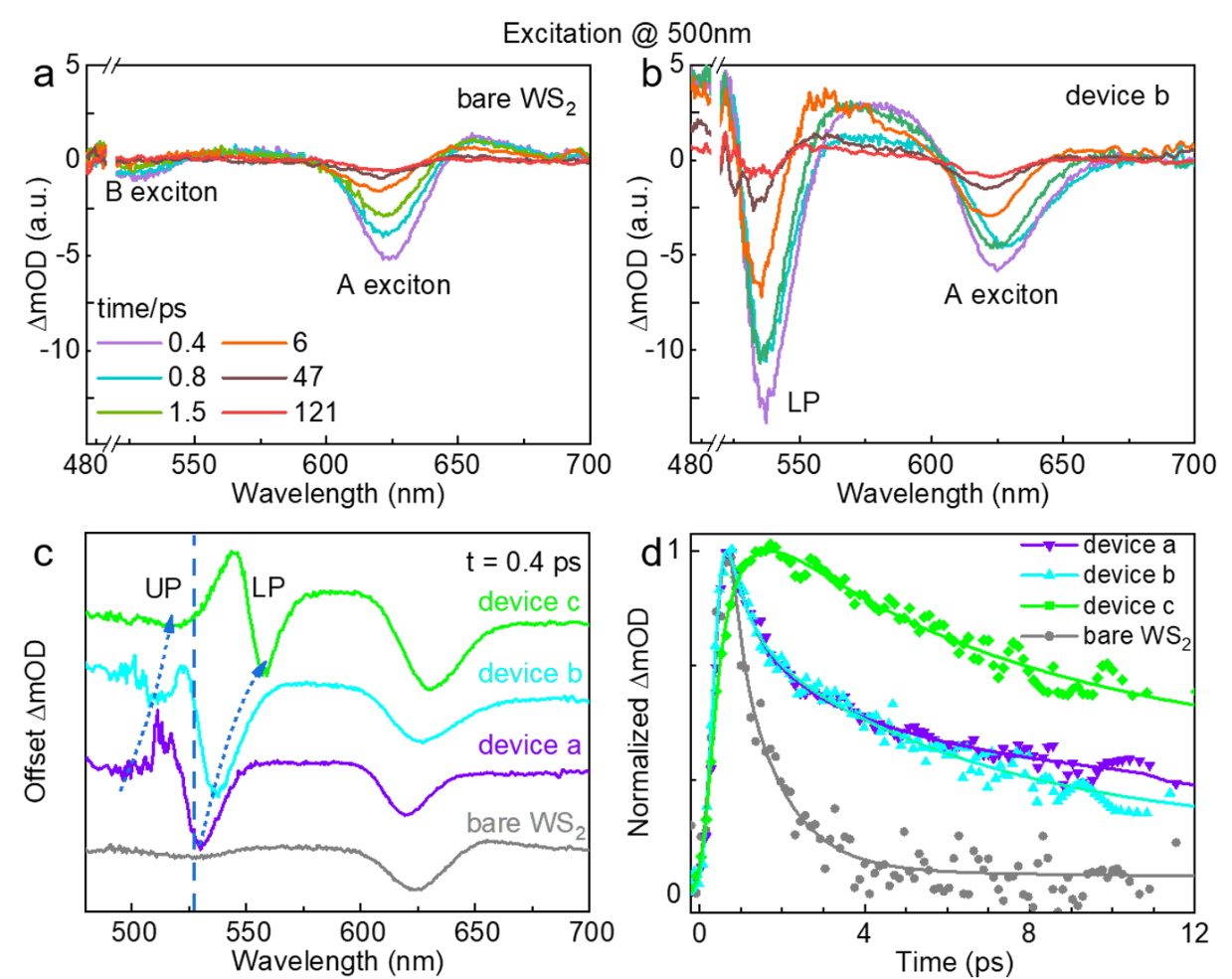


**Figure 2.** (a)-(b) Evolution of the TA spectra of bare monolayer WS_2_ and device a at different delay times (0.4 ps, 0.8 ps, 1.5 ps, 47 ps, and 121 ps). The region between 490 and 520 nm was removed because of distortions due to the high optical density of the sample. (c) TA spectra of monolayer WS_2_ and devices a-c at 0.4 ps. (d) Dynamics of the LP states of devices a-c and B excitons in monolayer WS_2_. The excitation wavelength is at 500 nm.

Next, to reveal the cavity-induced modulation on the PL emission of the A exciton, TA measurements were performed on devices d-h under 500 nm excitation (**Figure S5**). Since the cavity modes of devices e-g have a good overlap with the A exciton resonance, their initial TA spectra at 0.4 ps are first analyzed and shown in **Figure 3**a. Compared with bare monolayer WS_2_, two distinctive GSB signals associating with the hybrid cavity mode and A exciton states, appear with an anti-crossing behavior with the cavity length increasing, which is consistent with the signature of strong coupling more clearly. A Rabi splitting up to 110 meV can be extracted as shown in Figure S6b. Furthermore, by comparing the peak position of the LP states in TA spectra and the PL emission (Figure 1d) in devices e-g, the PL emission can be more clearly related to the LP state. Whereas, the lifetime of the LP states is not noticeably different from that of bare monolayer WS_2_. As shown in Figure 3b, the plots show that the LP states process a similar lifetime as the bleaching recovery of the A exciton, which indicates that the relaxation process of the LP states coupled with A excitons was not significantly modified. It is also the reason why the cavity-induced PL enhancement factor is lower in the strong coupling devices with A exciton. In addition, the GSB peak of device h is tuned to 720 nm shown in Figure S7, which corresponds to that the PL emission of device h is also tuned to 720 nm.


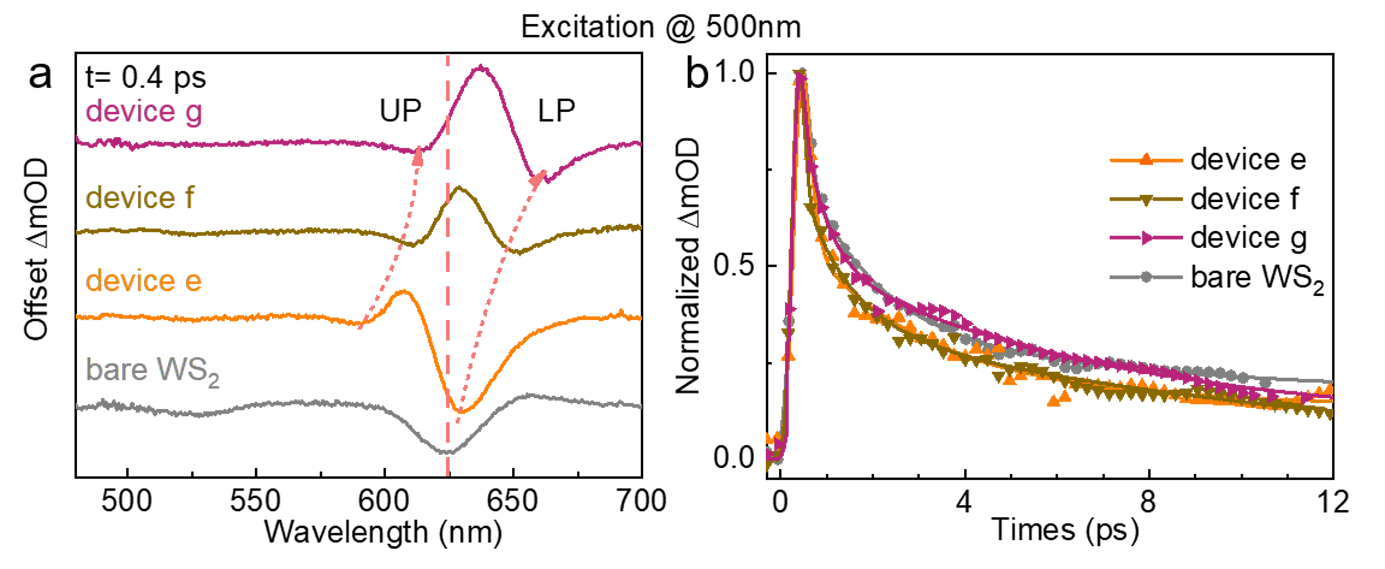


**Figure 3.** (a) TA spectra of monolayer WS_2_ and devices e-g at 0.4 ps. (b) Dynamics of the LP states of devices e-g and A exciton of monolayer WS_2_. The excitation wavelength is at 500 nm.

Finally, we turn to the TA study on device d, in which an unusual PL emission peak appeared on the higher energy side of the A exciton. In general, PL emission from excited states is difficult to obtain due to Kasha’s rule [42]. Specifically, for high-energy excited states, the population density is usually lower and the radiation dipole moment is weak, making it a difficult task to reveal these states in static optical measurements. Thus, TA experiments were further performed on device d and the result is shown in **Figure 4**a. Compared with the control sample (Figure 2a), one obvious difference is that an additional GSB signal appears at 570 nm between the A and B exciton states.

Indeed, it has been demonstrated that there are a series of internal exciton states in monolayer WS_2_ on the analogy of the Rydberg energy levels in the hydrogen atom, which can also be characterized by the principal quantum number (*n* = 1, 2, 3 …) and the *s*, *p, d* orbitals with quantized angular momentum as the insert picture shown in Figure 4a [9]. However, due to the weak oscillator strength and short lifetime of the high-energy Rydberg state, their emission is much weaker than that of 1s state. Even though for high-quality monolayer WS_2_ encapsulated in h-BN film, the 2s, 3s, and 4s excitons have been well confirmed by utilizing photoluminescence excitation spectroscopy. In particular, the 2s excited state of A exciton in monolayer WS_2_ is assigned at 2.17 eV [13]. Thus, we would like to associate the additional GSB signal at 570 nm with the 2s excited states, which arise from the cavity-induced enhancement. As a result, the unusual PL emission peak at 578 nm can also be explained by the enhanced population density of 2s states as revealed in the TA spectra. Another should be noted is that the energy level of 2s Rydberg state is unperturbed since the additional GSB signal peak coincided with the original position of 2s Rydberg state and not splitting. Hence, we suggest that the coupling between 2s Rydberg state and the cavity mode is in the weak coupling region, which is caused by the extremely weak oscillator strength of the Rydberg state exciton.

To ensure the additional GSB signal comes from the enhanced 2s Rydberg states, device d was further excited by a pump laser at 620 nm, in resonance with the A exciton. The TA spectra are reported in Figure 4b. The additional GSB signal peak at 570 nm can still be observed and is in good agreement with that under 500 nm excitation, which demonstrated that the additional state and the A exciton shared the same eigenstate. Hence, we confirm the additional GSB signal is the Rydberg state of A exciton. Finally, the extracted TA spectra at 6 ps excited under 500 and 620 nm, as well as the corresponding PL emission are compared in Figure 4c, in which the coincided additional GSB signal at 570 nm and the corresponding noticeable PL emission peak appeared at 578 nm clearly fingerprint of the cavity-enhanced 2s Rydberg states.





**Figure 4.** (a) - (b) TA spectra of device d at different delay times under 500 nm (a) and 620 nm (b) excitation respectively. The insert picture in (a) shows internal exciton states of monolayer WS_2_. (c) Comparison TA and PL spectra of device d. The PL spectrum of bare monolayer WS_2_ was shown by the dark gray solid line.

# 3. Conclusion

To conclude, we have demonstrated that PL emission of monolayer WS_2_ can be flexibly tailored in a broad visible wavelength range (490 nm – 720 nm) by constructing coupled WS_2_-Ag microcavity devices. In particular, the dynamics of the coupled exciton-photon states were investigated by using an ultrafast pump-probe approach. Strongly coupled hybrid states formed by the A/B exciton and the cavity photons were confirmed in TA spectra, respectively. For the devices strongly coupled with B exciton (high energy excited state), the bleaching signals of their LP states were significantly enhanced compared with that of the bare B exciton. Besides, the internal conversion pathway was blocked, leading to a longer lifetime of the LP states. Thus, the population in the LP states preferred to directly relax to the ground state without going through the A exciton state. The above two points can be responsible for the maximum enhancement factor of 25-fold for the PL emission from the LP states coupled with the B exciton. In contrast, for the devices strongly coupled with A exciton, the relaxation process of the hybrid states was not significantly regulated, resulting in a small PL emission enhancement factor. Finally, when the cavity mode is tuned to the higher energy side of the A excitons, a remarkable PL emission from the 2s Rydberg state is revealed and confirmed by the additional ground state bleaching in the TA spectra. The wide tunable emission peaks in the light-matter coupling devices with low dimensional TMDCs can pave the way for constructing highly efficient optoelectronic devices.

# 4. Methods

**Wet transfer method.** The wet transfer method mainly referred to Lu’s work [43]. First, 1.5 g of PVP (Alfa Aesar, average MW 58 000), 1.5 mL of NVP (J&K, 99.5%), and 0.75 mL of deionized water were mixed in 7ml of ethanol. Then the solution drops on the SiO_2_/Si substrate which adhered to the triangular monolayer WS_2_ flakes and spun at 1500 rpm for 1 min before it was baked at 75 °C for 1 min. Then the PVA (9 wt %, Alfa Aesar, 98−99% hydrolyzed, high molecular weight) was dropped and spun at 1500 rpm for 1 min. The PVP film can increase the adhesion between the WS_2_ flakes and the PVA film. Therefore, the triangular monolayer WS_2_ flakes (purchased from SixCarbon Technology ShenZhen) attached to the PVA/PVP film can be peeled off from the SiO_2_/Si substrate. After the PVA/PVP film was adhered to SiO_2_ film, it can be dissolved in deionized water for 1 h. Finally, the triangular monolayer WS_2_ flakes adhered to the bottom SiO_2_ film of the microcavity.

Besides, to confirm the monolayer WS_2_ was not destroyed during the top half of SiO_2_ film evaporation, the Raman spectra are compared in **Figure S2**. As can be seen, compared with the Raman characteristic peak of bare monolayer WS_2_ on SiO_2_/Ag film, the peak positions of monolayer WS_2_ after being embedded in microcavity were not changed, indicating that the evaporation of upper SiO_2_ film does not cause the deformation of monolayer WS_2_.

**Optical measurements.** The PL and Raman spectra of the samples were got from HORIBA T64000. All the samples were excited by the 473 nm laser. The TA spectra were measured by the pump-probe setup. The fundamental pulses (800 nm, 500 Hz, 35 fs) generated by a titanium sapphire laser system (Solstice, Spectra-Physics) were split into two laser beams. The 500 nm or 620 nm pump laser was generated after the stronger beam passed through an optical amplifier (TOPAS, Spectra-Physics). The size of the pump light is about 200 μm after focused by a lens. Another beam passed through a 2 mm sapphire to generate a broadband white light (450-800 nm) as the probe laser. The probe spot is about 5 μm under the focus of the objective, which is small enough to measure the monolayer WS_2_ flakes. After reflected from the sample, the probe light was collected by the same objective and sent to the spectrometer (Avantes-ULS2048CL-EVO). The mechanical delay stage (DL325, Newport) can manipulate the delay time of the pump and the probe light. Then the dispersion of TA spectra was all compensated by a chirp program.

Acknowledgments

The authors are grateful for the National Key Research and Development Program of China and the National Natural Science Foundation of China (NSFC) under Grants 21903035, 22073037, 21773087, 12074141, 12274168 and Jilin Provincial Science and Technology Development Project (20210509038RQ).

**Author contribution:**

All the authors have accepted responsibility for the entire content of this submitted manuscript and approved submission.

**Research funding:**

The authors are grateful for the National Key Research and Development Program of China and the National Natural Science Foundation of China (NSFC) under Grants 21903035, 22073037, 21773087, 12074141, 12274168 and Jilin Provincial Science and Technology Development Project (20210509038RQ).

**Conflict of interest statement:**

The authors declare no conflicts of interest regarding this article.

**References**

[1] Mak, K. F.; Lee, C.; Hone, J.; Shan, J.; Heinz, T. F., “Atomically Thin MoS_2_: A New Direct-Gap Semiconductor,” *Phys. Rev. Lett.*, vol. 105, pp. 1-4, 2010.

[2] Amani, M.; Lien, D. H.; Kiriya, D.; Xiao, J.; Azcatl, A.; Noh, J.; Madhvapathy, S. R.; Addou, R.; Santosh, K. C.; Dubey, M.; Cho, K.; Wallace, R. M.; Lee, S. C.; He, J. H.; Ager, J. W.; Zhang, X.; Yablonovitch, E.; Javey, A., “Near-unity photoluminescence quantum yield in MoS_2_,” *Science*, vol. 350, pp. 1065-1068, 2015.

[3] Wu, B.; Zheng, H. H.; Li, S. F.; Ding, J. N.; He, J.; Zeng, Y. J.; Chen, K. Q.; Liu, Z. W.; Chen, S. L.; Pan, A. L.; Liu, Y. P., “Evidence for moire intralayer excitons in twisted WSe_2_/WSe_2_ homobilayer superlattices,” *Light-Sci. Appl.*, vol. 11, pp. 1-8, 2022.

[4] Shin, D.; Huebner, H.; De Giovannini, U.; Jin, H.; Rubio, A.; Park, N., “Phonon-driven spin-Floquet magneto-valleytronics in MoS_2_,” *Nat. Commun.*, vol. 9, pp.1-8, 2018.

[5] Yuan, L.; Huang, L., Exciton dynamics and annihilation in WS_2_ 2D semiconductors. *Nanoscale*, 7, pp. 7402-7408, 2015.

[6] Zhao, W. J.; Ghorannevis, Z.; Chu, L. Q.; Toh, M. L.; Kloc, C.; Tan, P. H.; Eda, G., “Evolution of Electronic Structure in Atomically Thin Sheets of WS_2_ and WSe_2_,” *ACS Nano*, vol. 7, pp. 791-797, 2013.

[7] Ye, Z. L.; Cao, T.; O'Brien, K.; Zhu, H. Y.; Yin, X. B.; Wang, Y.; Louie, S. G.; Zhang, X., “Probing excitonic dark states in single-layer tungsten disulphide,” *Nature*, vol. 513, pp. 214-218, 2014.

[8] Hanbicki, A. T.; Currie, M.; Kioseoglou, G.; Friedman, A. L.; Jonker, B. T., “Measurement of high exciton binding energy in the monolayer transition-metal dichalcogenides WS_2_ and WSe_2_,” *Solid State Commun*, vol. 203, pp. 16-20, 2015.

[9] Chernikov, A.; Berkelbach, T. C.; Hill, H. M.; Rigosi, A.; Li, Y. L.; Aslan, O. B.; Reichman, D. R.; Hybertsen, M. S.; Heinz, T. F., “Exciton Binding Energy and Nonhydrogenic Rydberg Series in Monolayer WS_2_,” *Phys. Rev. Lett.*, vol. 113, pp. 1-5, 2014.

[10] Pandey, J.; Soni, A., “Unraveling biexciton and excitonic excited states from defect bound states in monolayer MoS_2_,” *Appl. Surf. Sci.*, vol. 463, pp. 52-57, 2019.

[11] Riche, F.; Braganca, H.; Qu, F. Y.; Lopez-Richard, V.; Xie, S. J.; Dias, A. C.; Marques, G. E., “Robust room temperature emissions of trion in darkish WSe_2_ monolayers: effects of dark neutral and charged excitonic states,” *J. Phys. Condens. Matter*, vol. 32, pp.1-10, 2020.

[12] Chen, S. Y.; Lu, Z. G.; Goldstein, T.; Tong, J. Y.; Chaves, A.; Kunstmann, J.; Cayalcante, L. S. R.; Wozniak, T.; Seifert, G.; Reichman, D. R.; Taniguchi, T.; Watanabe, K.; Smimov, D.; Yan, J., “Luminescent Emission of Excited Rydberg Excitons from Monolayer WSe_2_,” *Nano Lett.*, vol. 19, pp. 2464-2471, 2019.

[13] Hill, H. M.; Rigosi, A. F.; Roquelet, C.; Chernikov, A.; Berkelbach, T. C.; Reichman, D. R.; Hybertsen, M. S.; Brus, L. E.; Heinz, T. F., “Observation of Excitonic Rydberg States in Monolayer MoS_2_ and WS_2_ by Photoluminescence Excitation Spectroscopy,” *Nano Lett.*, vol. 15, pp. 2992-2997, 2015.

[14] Gu, J.; Walther, V.; Waldecker, L.; Rhodes, D.; Raja, A.; Hone, J. C.; Heinz, T. F.; Kena-Cohen, S.; Pohl, T.; Menon, V. M., “Enhanced nonlinear interaction of polaritons via excitonic Rydberg states in monolayer WSe_2_,” *Nat. Commun.*, vol. 12, pp. 1-7, 2021.

[15] Hertzog, M.; Wang, M.; Mony, J.; Borjesson, K., “Strong light-matter interactions: a new direction within chemistry. *Chem*,” *Soc. Rev.*, vol. 48, pp. 937-961, 2019.

[16] Yu, X. T.; Yuan, Y. F.; Xu, J. H.; Yong, K. T.; Qu, J. L.; “Song, J., Strong Coupling in Microcavity Structures: Principle, Design, and Practical Application” *Laser Photon.*, *Rev.* vol. 13, pp. 1-19, 2019.

[17] Piccione, B.; Aspetti, C. O.; Cho, C. H.; Agarwal, R., “Tailoring light-matter coupling in semiconductor and hybrid-plasmonic nanowires,” *Rep. Prog. Phys.*, vol. 77, pp. 1-20, 2014.

[18] Qian, Z. Y.; Shan, L. X.; Zhang, X. C.; Liu, Q.; Ma, Y.; Gong, Q. H.; Gu, Y., “Spontaneous emission in micro- or nanophotonic structures,” *PhotoniX*, vol. 2, pp. 1-35, 2021.

[19] Canet-Ferrer, J.; Prieto, I.; Munoz-Matutano, G.; Martinez, L. J.; Munoz-Camuniez, L. E.; Llorens, J. M.; Fuster, D.; Alen, B.; Gonzalez, Y.; Gonzalez, L.; Postigo, P. A.; Martinez-Pastor, J. P., “Excitation power dependence of the Purcell effect in photonic crystal microcavity lasers with quantum wires,” *Appl. Phys. Lett.*, vol. 102, pp. 1-4, 2013.

[20] Zang, C.; Liu, S.; Xu, M.; Wang, R.; Cao, C.; Zhu, Z.; Zhang, J.; Wang, H.; Zhang, L.; Xie, W.; Lee, C.-S., “Top-emitting thermally activated delayed fluorescence organic light-emitting devices with weak light-matter coupling,” *Light Sci. Appl.*, vol. 10, pp. 1-10, 2021.

[21] Liu, X. Z.; Galfsky, T.; Sun, Z.; Xia, F. N.; Lin, E. C.; Lee, Y. H.; Kena-Cohen, S.; Menon, V. M., “Strong light-matter coupling in two-dimensional atomic crystals.” *Nat. Photonics*, vol. 9, pp. 30-34, 2015.

[22] Schneider, C.; Glazov, M. M.; Korn, T.; Hoefling, S.; Urbaszek, B., “Two-dimensional semiconductors in the regime of strong light-matter coupling,” *Nat. Commun.*, vol. 9, pp. 1-9, 2018.

[23] Goncalves, P. A. D.; Stenger, N.; Cox, J. D.; Mortensen, N. A.; Xiao, S., “Strong Light-Matter Interactions Enabled by Polaritons in Atomically Thin Materials,” *Adv. Opt. Mater.*, vol, 8, pp. 1-25, 2020.

[24] Kleemann, M.-E.; Chikkaraddy, R.; Alexeev, E. M.; Kos, D.; Carnegie, C.; Deacon, W.; de Pury, A. C.; Große, C.; de Nijs, B.; Mertens, J.; Tartakovskii, A. I.; Baumberg, J. J., “Strong-coupling of WSe_2_ in ultra-compact plasmonic nanocavities at room temperature,” *Nat. Commun.*, vol. 8, pp. 1-7, 2017.

[25] Liu, T. Y.; Wang, H.; Song, M. S.; Zhao, L. Y.; Hu, Z. F.; Wang, H. Y., “Dynamics of Spin-Dependent Polariton-Polariton Interactions in Two-Dimensional Layered Halide Organic Perovskite Microcavities,” *Laser Photon. Rev.*, vol. 16, pp. 1-9, 2022.

[26] Wang, H.; Wang, H.-Y.; Bozzola, A.; Toma, A.; Panaro, S.; Raja, W.; Alabastri, A.; Wang, L.; Chen, Q.-D.; Xu, H.-L.; De Angelis, F.; Sun, H.-B.; Zaccaria, R. P., ‘Dynamics of Strong Coupling between J-Aggregates and Surface Plasmon Polaritons in Subwavelength Hole Arrays,” *Adv. Funct. Mater.*, vol. 26, pp. 6198-6205, 2016.

[27] Wang, H.; Wang, H.-Y.; Sun, H.-B.; Cerea, A.; Toma, A.; De Angelis, F.; Jin, X.; Razzari, L.; Cojoc, D.; Catone, D.; Huang, F.; Zaccaria, R. P., “Dynamics of Strongly Coupled Hybrid States by Transient Absorption Spectroscopy,” *Adv. Funct. Mater.*, vol. 28, pp. 1-28, 2018.

[28] Wang, H.; Toma, A.; Wang, H.-Y.; Bozzola, A.; Miele, E.; Haddadpour, A.; Veronis, G.; De Angelis, F.; Wang, L.; Chen, Q.-D.; Xu, H.-L.; Sun, H.-B.; Zaccaria, R. P., “The role of Rabi splitting tuning in the dynamics of strongly coupled J-aggregates and surface plasmon polaritons in nanohole arrays,” *Nanoscale*, vol. 8, pp. 13445-13453, 2016.

[29] Ye, Y.; Wong, Z. J.; Lu, X.; Ni, X.; Zhu, H.; Chen, X.; Wang, Y.; Zhang, X., “Monolayer excitonic laser,” *Nat. Photonics*, vol. 9, pp. 733-737, 2015.

[30] Wu, S.; Buckley, S.; Schaibley, J. R.; Feng, L.; Yan, J.; Mandrus, D. G.; Hatami, F.; Yao, W.; Vučković, J.; Majumdar, A.; Xu, X., “Monolayer semiconductor nanocavity lasers with ultralow thresholds,” *Nature*, vol. 520, pp. 69-72, 2015.

[31] Zhao, L.-Y.; Wang, H.; Wang, H.-Y.; Zhou, Q.; Zhang, X.-L.; Cui, T.; Wang, L.; Liu, T.-Y.; Han, Y.-X.; Luo, Y.; Yue, Y.-Y.; Song, M.-S.; Sun, H.-B., “Ultrafast modulation of valley dynamics in multiple WS_2_ − Ag gratings strong coupling system,” *PhotoniX*, vol. 3, pp. 1-20, 2022.

[32] Ding, B.; Zhang, Z.; Chen, Y.-H.; Zhang, Y.; Blaikie, R. J.; Qiu, M., “Tunable Valley Polarized Plasmon-Exciton Polaritons in Two-Dimensional Semiconductors,” *ACS Nano*, vol.13, pp. 1333-1341, 2019.

[33] Han, X.; Wang, K.; Jiang, Y.; Xing, X.; Li, S.; Hu, H.; Liu, W.; Wang, B.; Lu, P., “Controllable Plexcitonic Coupling in a WS_2_-Ag Nanocavity with Solvents,” *ACS Appl. Mater. Interfaces*, vol. 13, pp. 43554-43561, 2021.

[34] Rose, A.; Dunklin, J. R.; Zhang, H. Y.; Merlo, J. M.; van de Lagemaat, J., “Plasmon-Mediated Coherent Superposition of Discrete Excitons under Strong Exciton-Plasmon Coupling in Few-Layer MoS2 at Room Temperature,” *ACS Photonics*, vol. 7, pp. 1129-1134, 2020.

[35] Huang, J.; Akselrod, G. M.; Ming, T.; Kong, J.; Mikkelsen, M. H., “Tailored Emission Spectrum of 2D Semiconductors Using Plasmonic Nanocavities,” *ACS Photonics*, vol. 5, pp. 552-558, 2018.

[36] Li, S.-L.; Zhou, L.-D.; Deng, F.; Xiang, J.; Panmain, M. C.; Huang, H.-G.; Li, G.-C.; Chen, G.-D.; Lan, S., “Transverse-Electric-Polarized Polaritons Propagating in a WS_2_/Si3N4/Ag Heterostructure,” *Laser Photon. Rev.*, vol. 12, pp. 1-9, 2022.

~~[36]~~ [37] Pu, J.; Takenobu, T., “Monolayer Transition Metal Dichalcogenides as Light Sources. *Adv. Mater.*,” vol. 30, pp. 1-22, 2018.

[38] Bellessa, J.; Bonnand, C.; Plenet, J. C.; Mugnier, J., “Strong coupling between surface plasmons and excitons in an organic semiconductor,” *Phys. Rev. Lett.*, vol. 93, pp. 1-4, 2004.

[39] Lidzey, D. G.; Fox, A. M.; Rahn, M. D.; Skolnick, M. S.; Agranovich, V. M.; Walker, S., “Experimental study of light emission from strongly coupled organic semiconductor microcavities following nonresonant laser excitation,” *Phys. Rev. B*, vol. 65, pp. 1-10, 2002.

[40] Wang, Z.; Molina-Sánchez, A.; Altmann, P.; Sangalli, D.; De Fazio, D.; Soavi, G.; Sassi, U.; Bottegoni, F.; Ciccacci, F.; Finazzi, M.; Wirtz, L.; Ferrari, A. C.; Marini, A.; Cerullo, G.; Dal Conte, S., “Intravalley Spin–Flip Relaxation Dynamics in Single-Layer WS2,” *Nano Lett.*, Vol. 18, pp. 6882-6891, 2018.

[41] Rose, A. H.; Aubry, T. J.; Zhang, H.; Vigil-Fowler, D.; van de Lagemaat, J., “Ultrastrong Coupling Leads to Slowed Cooling of Hot Excitons in Few-Layer Transition-Metal Dichalcogenides,” *J. Phys. Chem. C*, vol. 126, pp. 8710-8719, 2022.

[42] Xu, Y. W.; Xu, P.; Hu, D. H.; Ma, Y. G., “Recent progress in hot exciton materials for organic light-emitting diodes,” *Chem. Soc. Rev.*, vol. 50, pp. 1030-1069, 2021.

[43] Lu, Z. X.; Sun, L. F.; Xu, G. C.; Zheng, J. Y.; Zhang, Q.; Wang, J. Y.; Jiao, L. Y., “Universal Transfer and Stacking of Chemical Vapor Deposition Grown Two-Dimensional Atomic Layers with Water-Soluble Polymer Mediator,” *ACS Nano*, vol. 10, pp. 5237-5242, 2016.
